# Transcriptomic Analysis Identifies Differentially Expressed Genes (DEGs) Associated with Bolting and Flowering in Radish (*Raphanus sativus* L.)

**DOI:** 10.3389/fpls.2016.00682

**Published:** 2016-05-24

**Authors:** Shanshan Nie, Chao Li, Yan Wang, Liang Xu, Everlyne M. Muleke, Mingjia Tang, Xiaochuan Sun, Liwang Liu

**Affiliations:** National Key Laboratory of Crop Genetics and Germplasm Enhancement, Key Laboratory of Biology and Genetic Improvement of Horticultural Crops (East China), Ministry of Agriculture of China, College of Horticulture, Nanjing Agricultural UniversityNanjing, China

**Keywords:** *Raphanus sativus* L., bolting and flowering, RNA-Seq, hormone signaling, differentially expressed genes (DEGs)

## Abstract

The transition of vegetative growth to bolting and flowering is an important process in the life cycle of plants, which is determined by numerous genes forming an intricate network of bolting and flowering. However, no comprehensive identification and profiling of bolting and flowering-related genes have been carried out in radish. In this study, RNA-Seq technology was applied to analyze the differential gene expressions during the transition from vegetative stage to reproductive stage in radish. A total of 5922 differentially expressed genes (DEGs) including 779 up-regulated and 5143 down-regulated genes were isolated. Functional enrichment analysis suggested that some DEGs were involved in hormone signaling pathways and the transcriptional regulation of bolting and flowering. KEGG-based analysis identified 37 DEGs being involved in phytohormone signaling pathways. Moreover, 95 DEGs related to bolting and flowering were identified and integrated into various flowering pathways. Several critical genes including *FT, CO, SOC1, FLC*, and *LFY* were characterized and profiled by RT-qPCR analysis. Correlation analysis indicated that 24 miRNA-DEG pairs were involved in radish bolting and flowering. Finally, a miRNA-DEG-based schematic model of bolting and flowering regulatory network was proposed in radish. These outcomes provided significant insights into genetic control of radish bolting and flowering, and would facilitate unraveling molecular regulatory mechanism underlying bolting and flowering in root vegetable crops.

## Introduction

The developmental transition from vegetative growth to bolting and flowering is one of the most important traits in plant life cycle. Bolting and flowering time must be appropriately determined to ensure reproductive success under most favorable conditions (Amasino and Michaels, 2010; Srikanth and Schmid, 2011). Plants have evolved an intricate bolting and flowering genetic circuitry in response to various endogenous and environmental signals including development, age, plant hormones, photoperiod, and temperature (Fornara et al., 2010; Capovilla et al., 2015; Kazan and Lyons, 2015). Molecular and genetic regulation of flowering has been extensively studied in the model plant *Arabidopsis thaliana*. Five major flowering pathways including vernalization, photoperiod, autonomous, aging and gibberellin (GA) pathways have been identified to govern bolting and flowering time (Amasino and Michaels, 2010; Fornara et al., 2010; Srikanth and Schmid, 2011), and a number of flowering-related genes involved in these pathways have been isolated and characterized in *Arabidopsis* (Fornara et al., 2010; Srikanth and Schmid, 2011).

The signals from flowering pathways converge on several floral pathway integrators such as *FLOWERING LOCUS T* (*FT*), *SUPPRESSOR OF OVEREXPRESSION OF CO1* (*SOC1*) and *LEAFY* (*LFY*), which are integrated into the genetic networks of flowering (Moon et al., 2005; Parcy, 2005). Among these integrators, the florigen gene *FT* is a central node of floral transition, whose transcriptional expression is positively regulated by *CONSTANS* (*CO*) encoding a putative zinc finger transcription factor (Suárez-López et al., 2001), while it is negatively regulated by *FLOWERING LOCUS C* (*FLC*), a flowering repressor encoding a MADS-box transcription factor (Michaels and Amasino, 1999). Different environmental factors affect plant flowering by modulating the expression of floral integrators and stimulating changes in plant hormone levels (Yaish et al., 2011; Riboni et al., 2014; Kazan and Lyons, 2015). Increasing evidences have revealed the connections between flowering time and plant hormones including salicylic acid (SA), jasmonic acid (JA), GA, abscisic acid (ABA) and auxin (Davis, 2009; Kazan and Lyons, 2015). The effects of phytohormone signaling on flowering, particularly GA pathway, have been extensively described in *Arabidopsis* (Mutasa-Göttgens and Hedden, 2009). Therefore, understanding the roles of flowering-related genes and crosstalk between diverse genetic pathways is fundamental for elucidating the regulatory mechanisms underlying bolting and flowering in plants.

RNA sequencing (RNA-Seq), a powerful strategy for global discovery of functional genes, has provided a better qualitative and quantitative description of gene expressions under certain conditions in many plant species (Lister et al., 2009; Wang et al., 2009a). Digital gene expression (DGE) tag profiling is a revolutionary approach for identifying differentially expressed genes (DEGs) in diverse plant tissues, organs and developmental stages (Bai et al., 2013; Zhang et al., 2014a; Zhu et al., 2015). Moreover, RNA-Seq combined with DGE profiling has been employed for flowering-related gene discovery and expression analysis in some species such as bamboo (Gao et al., 2014), *Lagerstroemia indica* (Zhang et al., 2014b), sweetpotato (Tao et al., 2013) and litchi (Zhang et al., 2014c). However, to our knowledge, there are no studies on global expression profile analysis of bolting and flowering-related genes in radish (*Raphanus sativus* L.).

Radish (2*n* = 2*x* = 18), belonging to Brassicaceae family, is an important annual or biennial root vegetable crop worldwide. Premature bolting is a seriously destructive problem and results in poor root growth and the reduced harvest during radish production, especially in spring. Appropriate timing of bolting and flowering is significant for reproductive success at suitable conditions, as well as preventing the premature bolting in radish. Progress on bolting and flowering time control (Fornara et al., 2010; Srikanth and Schmid, 2011), especially in *Arabidopsis*, has provided a solid foundation and reference for identifying numerous functional genes during radish bolting and flowering. Recently, the transcriptomes from radish roots and leaves have been assembled and analyzed (Wang et al., 2013; Zhang et al., 2013; Xu et al., 2015). Moreover, a list of microRNAs (miRNAs) and functional genes related to bolting and flowering were successfully isolated from late-bolting radish based on transcriptomic datasets (Nie et al., 2015). Therefore, to further identify the DEGs involved in bolting and flowering regulation is of importance for understanding the genetic regulatory network of bolting and flowering in radish.

In this study, to investigate the gene expression patterns during the transition of vegetative growth to bolting stage in radish, using the late bolting radish advanced inbred line as material, two DGE libraries were constructed and sequenced with RNA-Seq technology. The aims were to comprehensively identify DEGs involved in bolting and flowering regulatory network and to explore their roles in determining radish bolting and flowering time. Expression patterns of several critical DEGs related to bolting and flowering were validated by quantitative real-time PCR (RT-qPCR) analysis. Finally, to characterize the bolting and flowering-related genes and miRNAs in flowering pathways, a putative miRNA-DEG-based model of bolting and flowering regulatory network was put forward in radish. These results could provide significant insights into the molecular mechanism underlying bolting and flowering regulation in radish and other root vegetable crops.

## Materials and methods

### Plant materials

The late bolting radish advanced inbred line ‘NAU-LU127’, which was self-pollinated for more than 20 generations, was used in this study. The genetic background and structure of this line are stable and highly homozygous. After surface-sterilization, the seeds were sowed and grew in a growth chamber with 16 h light at 25°C and 8 h darkness at 16°C. The radish leaves used for DGE sequencing and RT-qPCR analysis were separately collected at two different developmental stages: vegetative stage (VS) and reproductive stage (RS), with three biological replicates. Each sample was collected at two developmental stages from three randomly selected individual plants, respectively. All the samples were immediately frozen in liquid nitrogen and stored at −80°C until use.

### DGE library construction and illumina sequencing

Total RNA from radish leaves at vegetative stage and reproductive stage was individually extracted using Trizol® Reagent (Invitrogen) following the manufacturer's instructions. The equivalent quantity of total RNA from three replicates was pooled and used for library preparation and sequencing. Two cDNA libraries named NAU-VS and NAU-RS were constructed and sequenced according to the previously reported method (Xu et al., 2015). The library construction and Illumina sequencing were performed using HiSeq™ 2500 platform at Beijing Genomics Institute (BGI, Shenzhen, China). The RNA-Seq data were deposited in NCBI Sequence Read Archive (SRA, http://www.ncbi.nlm.nih.gov/Traces/sra/) with accession numbers of SRX1671036 (NAU-VS) and SRX1671054 (NAU-RS).

### Data processing and expression analysis of DEGs

The raw reads were primarily produced for data processing. After filtering low quality reads, adaptor sequences and reads containing ploy-N, the clean reads were obtained. These clean reads were then matched to the radish reference sequences which contained the public radish genomic survey sequences (GSS) and expressed sequence tag (EST) sequences and leaf transcriptome sequences from ‘NAU-LU127’ (Nie et al., in press) with no more than two mismatches. These sequences from radish leaf transcriptome have been deposited in NCBI Transcriptome Shotgun Assembly (TSA, http://www.ncbi.nlm.nih.gov/genbank/tsa/) database under the accession number GEMG00000000.

To screen the DEGs between two DGE libraries, the expression level of each transcript is calculated using RPKM (Reads Per kb per Million reads) method (Mortazavi et al., 2008). Prior to differential gene expression analysis, the read counts of each transcript were adjusted by edgeR program package (Robinson et al., 2010) through one scaling normalized factor. Trimmed Mean of M values (TMM), an appropriate normalization method implemented in the edgeR package (Robinson and Oshlack, 2010; Robinson et al., 2010), was employed to obtain the normalized read counts. The differential expression analysis of two libraries was performed using the DEGSeq R package 1.20.0 (Wang et al., 2010). Subsequently, the false discovery rate (FDR) was used to determine *P*-value threshold in multiple testing (Benjamini et al., 2001). A strict algorithm was used to further perform DEG identification according to the previous reports (Audic and Claverie, 1997). The absolute value of log_2_Ratio (NAU-RS/NAU-VS) ≥ 1, *P* < 0.05 and FDR ≤ 0.001 were used as threshold for judging the significance of gene expression difference. The cluster analysis of gene expression patterns was performed with cluster software and Java Treeview software (Saldanha, 2004).

### Functional annotation and enrichment analysis of DEGs

To investigate the biological function and involvement in functional pathways, all the identified transcripts were mapped to Gene Ontology (GO) and Kyoto Encyclopedia of Genes and Genomes (KEGG) database. For GO annotation, the unique transcripts were subjected to BLASTX searching against the NCBI Nr database using the *E* < 10^−5^. Then the Blast2GO (Conesa et al., 2005) and WEGO software (Ye et al., 2006) were used to obtain GO annotations and functional classifications. GO enrichment analysis of DEGs was implemented by the GOseq R package (Young et al., 2010). KOBAS software (Xie et al., 2011) was used to test the statistical enrichment of DEGs in KEGG pathways. The significantly enriched functional terms and pathways were identified using the criterion of a Bonferroni-corrected *P* ≤ 0.05.

### RT-qPCR analysis

Total RNAs from radish leaves were isolated and obtained as described above. RT-qPCR was performed on an iCycler Real-Time PCR Detection System (Bio-Rad, USA) with three replications according to previous reports (Nie et al., 2015; Xu et al., 2015). All PCR reactions were carried out in a total volume of 20 μL with *RsActin* gene as the internal control (Xu et al., 2012). The relative gene expression levels were calculated using 2${}^{-\Delta \Delta C_{\text{T}}}$ method (Livak and Schmittgen, 2001). The specific PCR primers were designed using Beacon Designer 7.0 (Premier Biosoft International, USA) and listed in Table S7.

## Results

### DGE library sequencing and data analysis

To obtain global unique sequences from radish leaves, *de novo* assembly and analysis of transcriptome prepared from radish leaves of ‘NAU-LU127’ were carried out using Illumina RNA-Seq technology. Totally 111,167 contigs and 53,642 unigenes were generated from the radish leaf transcriptome (Nie et al., in press). The available dataset of radish leaf transcriptome integrating with the available radish GSS and EST sequences released in NCBI database enriched the radish reference sequences for DEG identification during radish bolting and flowering.

**Figure 1:**
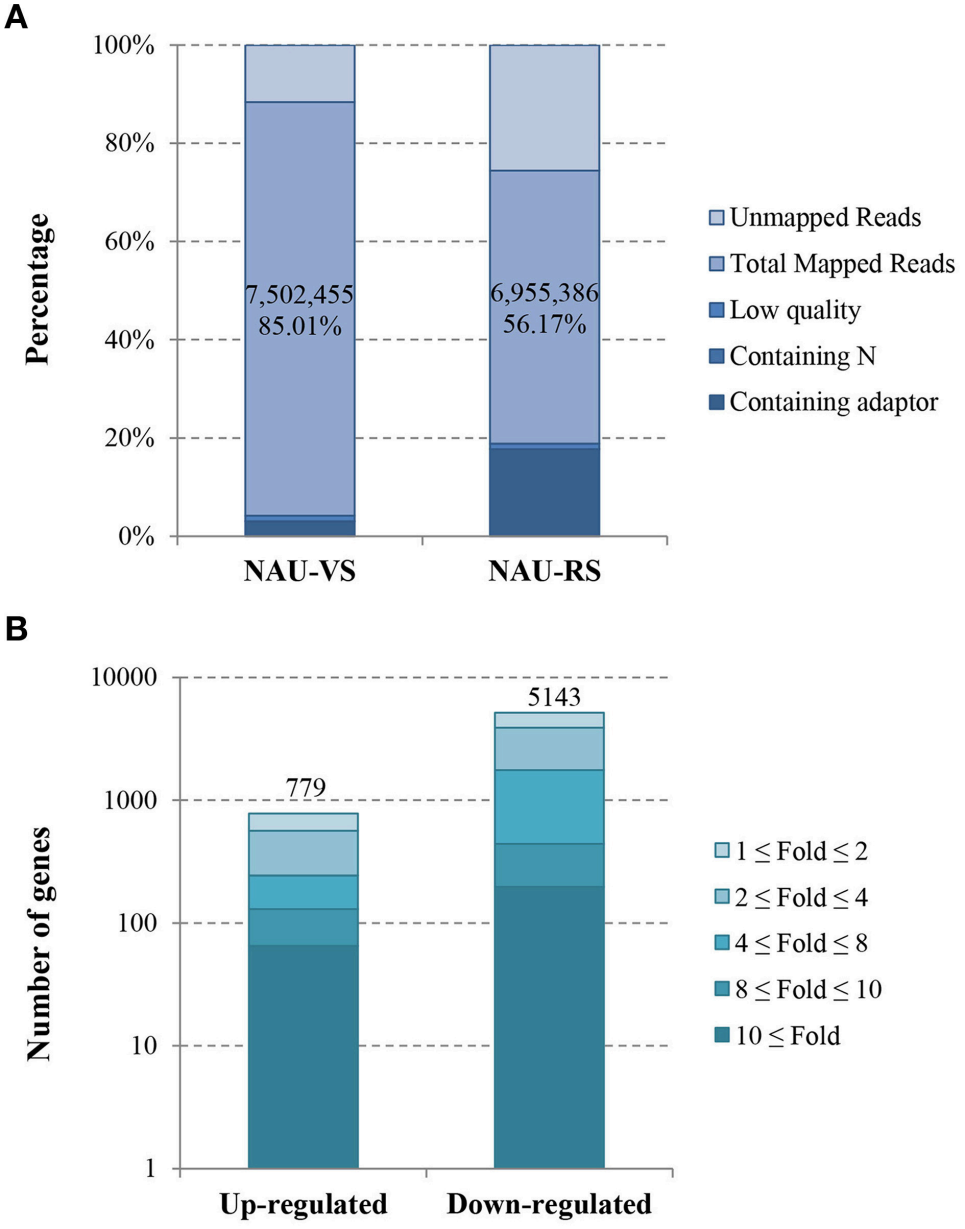
**Overview of the sequencing and mapping (A) and the identified DEGs (B) in NAU-VS and NAU-RS libraries**.

In this study, two DGE libraries from leaves of radish advanced inbred line ‘NAU-LU127’ at vegetative and reproductive stages were constructed and sequenced by Illumina HiSeq™ 2500 platform, respectively. As a result, 8,825,790 and 12,382,793 raw reads were obtained in NAU-VS and NAU-RS libraries, respectively (Table 1; Figure 1A). After removing adaptor sequences and low quality reads, 8,541,912 and 10,154,256 clean reads were generated in the two libraries (Table 1). These clean reads were then mapped to the radish reference sequences, resulting in the generation of 87.83% (7,502,455 reads) and 68.50% (6,955,386 reads) matched reads in NAU-VS and NAU-RS libraries, respectively. For the variation of clean reads mapping percentage between two libraries, it may arise from the sample differences and the specific pre-processing of obtained reads (Oshlack et al., 2010). The more mapped reads from NAU-VS library implied that some stage-specific genes may be expressed only at vegetative stage and differentially expressed between these two DGE libraries. Further analysis revealed that 4,124,632 reads (48.29 %) in NAU-VS library and 5,200,880 reads (51.22 %) in NAU-RS library were uniquely matched (Table 1).

**Table 1:** **Summary of DGE sequencing and mapped reads**.

**Summary**	**NAU-VS**		**NAU-RS**	
	**Reads number**	**Percent (%)**	**Reads number**	**Percent (%)**
Raw reads	8,825,790	100.00	12,382,793	100.00
Clean reads	8,541,912	100.00	10,154,256	100.00
Total base pairs	418,553,688	100.00	497,558,544	100.00
Total mapped reads	7,502,455	87.83	6,955,386	68.50
Perfect match	6,087,824	71.27	5,699,760	56.13
<=2bp mismatch	1,414,631	16.56	1,255,626	12.37
Unique match	4,124,632	48.29	5,200,880	51.22
Multi-position match	3,377,823	39.54	1,754,506	17.28
Unmapped reads	1,039,457	12.17	3,198,870	31.50

### Identification and functional enrichment analysis of DEGs

The transcript abundance of each gene from two DGE libraries was calculated and analyzed by RPKM method. The threshold of |log_2_Ratio| ≥ 1 and FDR ≤ 0.001 were further used to determine the significantly DEGs. A total of 5922 significantly DEGs including 779 up-regulated and 5143 down-regulated genes were obtained from NAU-VS and NAU-RS libraries (Table S1; Figure 1B).

**Figure 2:**
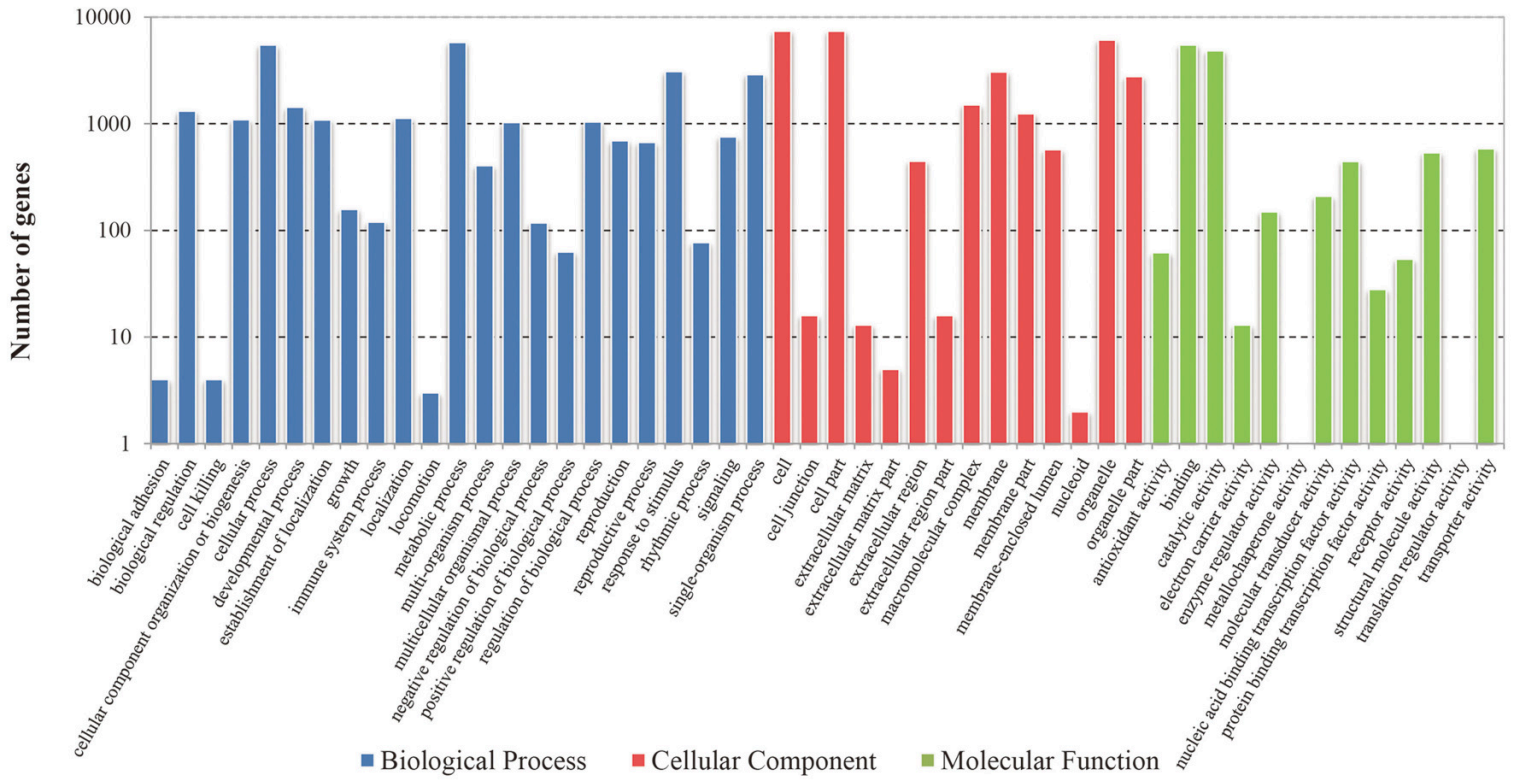
**The GO classification of identified DEGs in radish**.

To better classify the functions of these identified DEGs, GO classification and enrichment analysis were carried out in this study. These DEGs were categorized into three main GO categories including 23 biological processes, 14 cellular components and 13 molecular functions (Figure 2). Functional enrichment analysis revealed that 140 GO terms were significantly enriched with a Bonferroni-corrected *P* ≤ 0.05 (Table S2). The terms of “metabolic process” (GO: 0008152) and “organic substance metabolic process” (GO: 0071704) were the dominant groups in biological processes; “cell” (GO: 0005623) and “cell part” (GO: 0044464) were the highly represented groups in the cellular components. For the molecular functions, a large proportion of genes were significantly enriched in “organic cyclic compound binding” (GO: 0097159) and “heterocyclic compound binding” (GO: 1901363) categories. Moreover, some enriched GO terms were related to plant flowering and meristem development, including “regulation of photoperiodism, flowering” (GO: 2000028), “regulation of timing of meristematic phase transition” (GO: 0048506), “meristem maintenance” (GO: 0010073), “meristem growth” (GO: 0035266), “meristem development” (GO: 0048507) and “flower development” (GO: 0009908) (Table S2).

To further understand the putative active biological pathways, all the identified DEGs were mapped to KEGG database by BLASTx with *E* ≤ 10^−5^ and *Q* ≤ 1. As a result, 5922 DEGs were successfully assigned to 128 KEGG pathways (Table S3). The dominant pathway was “Metabolic pathways,” followed by “Biosynthesis of secondary metabolites,” “Ribosome,” and “Plant hormone signal transduction.” Moreover, 17 pathways were significantly enriched (*Q* ≤ 0.05; Table 2), including “Circadian rhythm-plant” (ko04712), “Plant hormone signal transduction” (ko04075), “Photosynthesis” (ko00195), “Ribosome” (ko03010) and “Vitamin B6 metabolism” (ko00750).

**Table 2:** **The significantly enriched pathways for DEGs in radish**.

**Pathway**	**Pathway ID**	**Number of DEGs**	***P*-value**	***Q*-value**
Amino sugar and nucleotide sugar metabolism	ko00520	142 (1.89%)	1.65E-03	1.76E-02
Aminoacyl-tRNA biosynthesis	ko00970	71 (0.95%)	4.91E-06	2.09E-04
Carbon fixation in photosynthetic organisms	ko00710	104 (1.38%)	1.43E-03	1.67E-02
Circadian rhythm-plant	ko04712	101 (1.34%)	2.33E-03	1.50E-02
Histidine metabolism	ko00340	23 (0.31%)	8.10E-04	1.04E-02
Lysine biosynthesis	ko00300	24 (0.32%)	1.29E-04	3.32E-03
Other types of O-glycan biosynthesis	ko00514	13 (0.17%)	4.60E-03	4.20E-02
Pentose phosphate pathway	ko00030	56 (0.75%)	2.61E-04	3.83E-03
Photosynthesis	ko00195	115 (1.53%)	2.69E-04	3.83E-03
Photosynthesis-antenna proteins	ko00196	36 (0.48%)	6.50E-05	2.08E-03
Plant hormone signal transduction	ko04075	394 (5.25%)	7.14E-03	3.27E-02
Porphyrin and chlorophyll metabolism	ko00860	68 (0.91%)	2.56E-06	1.64E-04
Protein export	ko03060	64 (0.85%)	3.02E-03	2.98E-02
Ribosome	ko03010	504 (6.71%)	2.25E-04	3.83E-03
RNA degradation	ko03018	156 (2.08%)	3.70E-07	4.73E-05
RNA transport	ko03013	240 (3.2%)	2.62E-04	3.83E-03
Vitamin B6 metabolism	ko00750	16 (0.21%)	5.85E-03	4.99E-02

### DEGs involved in hormone signal transduction pathway

**Figure 3:**
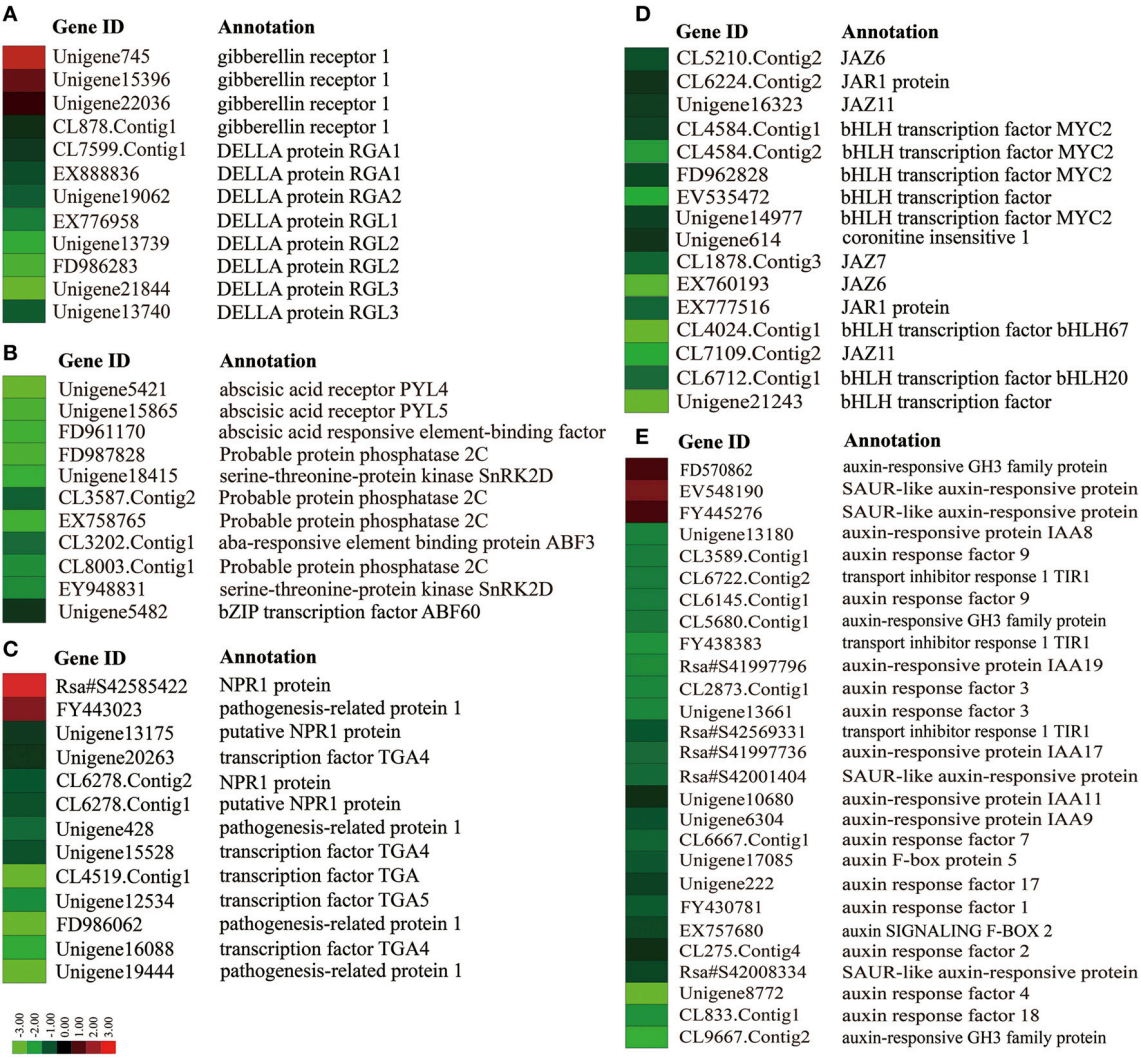
**Heat map diagram of expression patterns for DEGs involved in some phytohormone signaling pathways, including GA (A), ABA (B), SA (C), JA (D), and auxin (E)**. Red and green colors indicate up- and down-regulated genes in NAU-RS library as compared with NAU-VS library, respectively.

In this study, pathway-based analysis showed that 37 DEGs representing 393 unique sequences were identified and involved in “Plant hormone signal transduction” (ko04075) pathway (Table 3; Table S4; Figure S1). These genes including *AUX1, TRANSPORT INHIBITOR RESPONSE 1* (*TIR1*), *AUXIN RESPONSE FACTOR* (*ARFs*), *GIBBERELLIN RECEPTOR 1* (*GID1*), and *CORONITINE INSENSITIVE 1* (*COI1*), participated in the regulation of several hormone homeostasis and flowering time (Davis, 2009; Kazan and Lyons, 2015). Enrichment analysis revealed that most of genes were involved in auxin, GA, ABA, JA, and SA signaling pathways (Figure 3). In GA signaling pathway, one down- and three up-regulated transcripts were related to GID1 protein, while eight down-regulated transcripts encoded DELLA protein (Figure 3A). For the process of JA signaling, two down-regulated transcripts encoded JAR1 protein and one down-regulated transcript encoded COI1 protein (Figure 3D). In auxin signaling pathway, 10 down-regulated transcripts belonged to *ARF* genes, while one up- and seven down-regulated transcripts encoded auxin-responsive proteins (Figure 3E). In addition, some DEGs related to other phytohormone biosynthesis were also identified, including zeatin biosynthesis (ko00908, three DEGs), carotenoid biosynthesis (ko00906, four DEGs), cysteine and methionine metabolism (ko00270, seven DEGs), brassinosteroid biosynthesis (ko00905, eight DEGs), and phenylalanine metabolism (ko00360, three DEGs) (Table 3; Figure S1).

**Table 3:** **The identified DEGs involved in hormone signal transduction pathway in radish**.

**Gene entry**	**Gene name**	**Signaling pathway**	**Annotation**
K13946	*AUX1*	Auxin	Auxin influx carrier
K14485	*TIR1*	Auxin	Transport inhibitor response 1 TIR1
K14484	*AUX/IAA*	Auxin	Auxin-responsive protein IAA
K14486	*ARF*	Auxin	Auxin response factor
K14487	*GH3*	Auxin	Auxin-responsive GH3 family protein
K14488	*SAUR*	Auxin	SAUR-like auxin-responsive protein
K14489	*CRE1*	Cytokinin	Cytokinin receptor
K14491	*B-ARR*	Cytokinin	Two-component response regulator ARR-B family
K14492	*A-ARR*	Cytokinin	Two-component response regulator ARR-A family
K14493	*GID1*	Cytokinin	Gibberellin receptor 1
K14494	*DELLA*	Cytokinin	DELLA protein
K14496	*PYR/PYL*	Abscisic acid	Abscisic acid receptor PYL
K14497	*PP2C*	Abscisic acid	Probable protein phosphatase 2C
K14498	*SnRK2*	Abscisic acid	Serine-threonine-protein kinase SnRK2
K14432	*ABF*	Abscisic acid	Abscisic acid responsive element-binding factor
K14509	*ETR*	Ethylene	Ethylene response sensor
K14510	*CTR1*	Ethylene	Two-component response regulator ARR-A family
K14512	*MPK6*	Ethylene	Mitogen-activated protein kinase 6
K14513	*EIN2*	Ethylene	Ethylene-insensitive protein 2
K14515	*EBF1/2*	Ethylene	EIN3-binding F-box protein
K14514	*EIN3*	Ethylene	Ethylene-insensitive protein 3
K14517	*ERF1/2*	Ethylene	Ethylene-responsive transcription factor
K13416	*BAK1*	Brassinosteroids	Brassinosteroid insensitive 1-associated receptor kinase 1
K13415	*BRI1*	Brassinosteroids	Protein brassinosteroid insensitive 1
K14500	*BSK*	Brassinosteroids	BR-signaling kinase
K14501	*BSU1*	Brassinosteroids	BSU1 protein
K14502	*BIN2*	Brassinosteroids	Brassinosteroid insensitive protein2
K14503	*BZR1/2*	Brassinosteroids	BZR1 protein
K14504	*TCH4*	Brassinosteroids	Xyloglucan:xyloglucosyl transferase TCH4
K14505	*CYCD3*	Brassinosteroids	Cyclin D3, plant
K14506	*JAR1*	Jasmonate	JAR1 protein
K13463	*COI1*	Jasmonate	Coronitine insensitive 1
K13464	*JAZ*	Jasmonate	JAZ protein
K13422	*MYC2*	Jasmonate	bHLH transcription factor MYC2
K14508	*NPR1*	Salicylic acid	NPR1 protein
K14431	*TGA*	Salicylic acid	Transcription factor TGA
K13449	*PR-1*	Salicylic acid	Pathogenesis-related protein 1

### DEGs involved in the transition of vegetative growth to bolting in radish

In this study, to identify DEGs during radish bolting and flowering, BLAST searching was performed and the putative functions of DEGs were assessed. A total of 95 DEGs representing 128 unique sequences related to bolting and flowering were identified (Table S5). The analysis of flowering pathways revealed that these genes were involved in five different flowering pathways including photoperiod, vernalization, autonomous, GA and aging pathways.

In the present study, some unigenes representing photoperiodic flowering genes were identified, including *AGAMOUS-LIKE 24* (*AGL24*), *APETALA2* (*AP2*), *CO, CELL GROWTH DEFECT FACTOR 1* (*CDF1*), and *CONSTITUTIVELY PHOTOMORPHOGENIC 1* (*COP1*; Table S5). Some genes related to circadian rhythm and light signaling pathway included *CIRCADIAN CLOCK ASSOCIATED 1* (*CCA1*), *CONSTANS-LIKE 1* (*COL1*), *CRYPTOCHROME* (*CRY2*), *TIMING OF CAB EXPRESSION 1* (*TOC1*), and *LATE ELONGATED HYPOCOTYL* (*LHY*; Table S5).

For the vernalization pathway, one down-regulated transcript (CL1584.Contig3) belonging to *FLC* homolog was found in this study (Table S5), which is a major flowering repressor and integrates the autonomous and vernalization pathways (Michaels and Amasino, 1999). Many genes involved in vernalization pathway including *FRIGIDA* (*FRI*), *FRIGIDA-like* (*FRL*), *FRIGIDA INTERACTING PROTEIN 2* (*FIP2*), *EMBRYONIC FLOWER 2* (*EMF2*), *VERNALIZATION 1* (*VRN1*), and *VERNALIZATION 2* (*VRN2*) were also identified and implicated in regulating the expression of *FLC*. Furthermore, *LUMINIDEPENDENS* (*LD*), *FPA, FVE*, and *FY* involved in autonomous pathway were also identified (Table S5).

Moreover, some putative genes for GA and aging pathways were also found in the present study (Table S5). The candidate genes involved in GA pathway comprised *GIGANTEA* (*GI*), *GNC, GA INSENSITIVE DWARF 1B* (*GID1B*), *DWARF AND DELAYED FLOWERING 1* (*DDF1*), and *REPRESSOR OF GA 1-3* (*RGA1-3*). The candidate genes related to aging pathway included *SQUAMOSA PROMOTER BINDING-LIKE PROTEIN 1* (*SPL1*), *SPL2, SPL3, SPL9, SPL13*, and *SPL15*. In addition, some floral integrators such as *FT* (FD571044), *SOC1* (CL4258.Contig1) and *LFY* (Unigene29702), were also identified in this study (Table S5).

### Expression profile analysis by RT-qPCR

**Figure 4:**
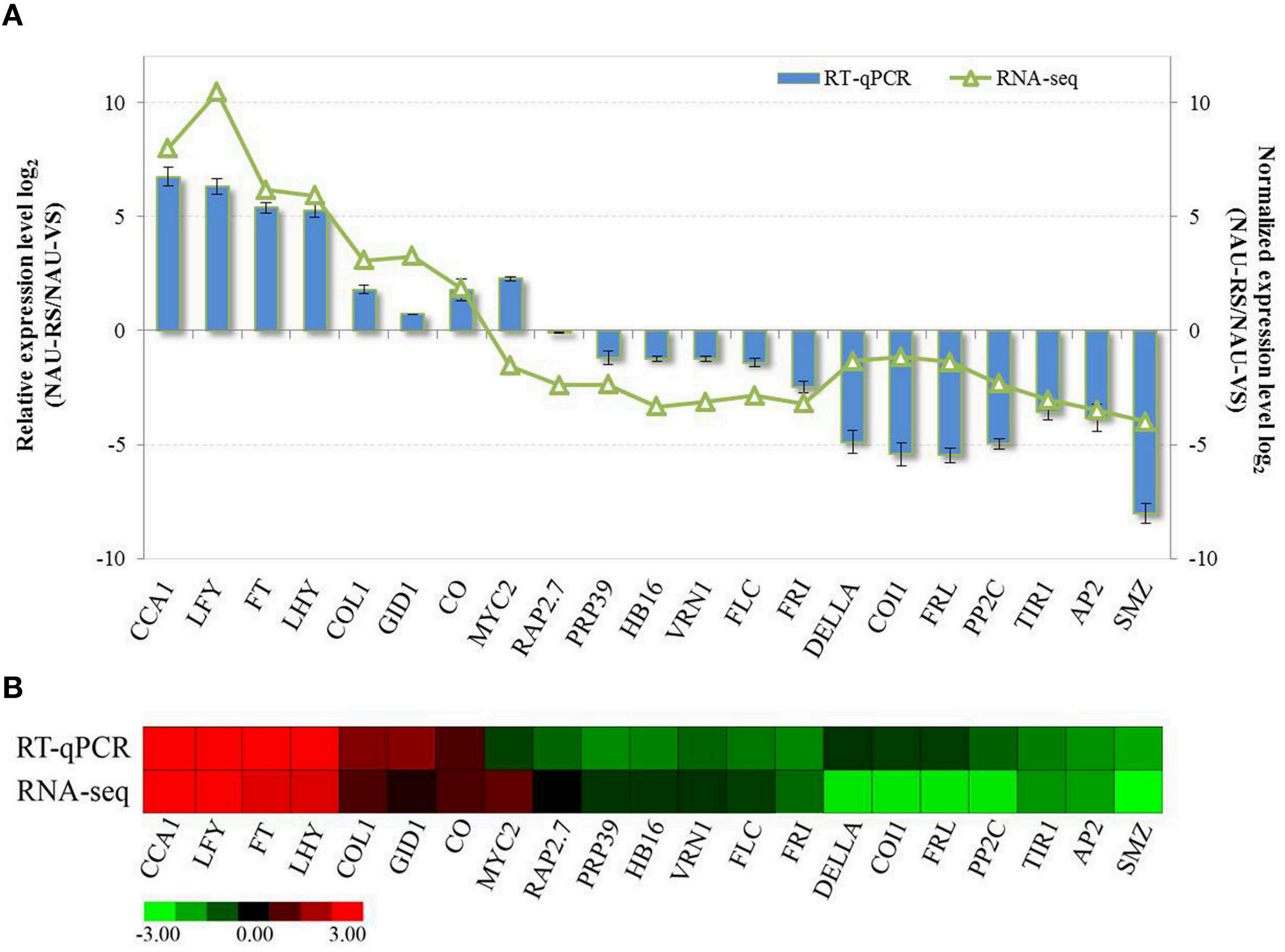
**The validation of expression levels of selected DEGs related to radish bolting and flowering. (A)** The relative expression levels of selected DEGs were compared with the transcript abundances from DGE sequencing. **(B)** Heat map diagram of expression patterns of DEGs in radish. Red and green colors in indicate up- and down-regulated genes in NAU-RS library as compared with NAU-VS library, respectively.

To validate the differential expression patterns of DEGs during radish bolting and flowering, totally 21 functional genes were randomly selected and subjected to RT-qPCR analysis. These selected genes included six genes related to hormone signaling and 15 genes related to bolting and flowering regulation. The relative expression levels of these genes between vegetative growth and reproductive stage were analyzed and compared (Figure 4). Further, comparative analysis revealed that these gene expression trends except *MYC2*-CL4584.Contig1 were in agreement with the transcript abundance changes by RNA-Seq (Figure 4), indicating the highly accuracy and quality of DGE sequencing.

### The regulatory network underlying bolting and flowering in radish

Considerable studies have revealed that some miRNAs regulating corresponding target genes played important roles in the transition from vegetative growth to bolting and flowering (Spanudakis and Jackson, 2014). In our recent study, several bolting and flowering-related miRNA-target gene pairs were identified and characterized in late-bolting radish (Nie et al., 2015). To better understand the genetic regulatory network of radish bolting and flowering, correlation analysis between the DEGs identified in the present study and bolting and flowering-related miRNAs previously reported (Nie et al., 2015) was performed. As expected, 24 miRNA-mRNA pairs including 16 miRNAs and 27 target DEGs were identified (Table S6). Among them, 19 miRNA-mRNA pairs showed negative correlations in expression patterns. Several DEGs including *AP2* (targeted by miR172), *VRN1* (targeted by miR5227), *PRP39* (targeted by miR6273), and *NF-YB3* (targeted by miR860), were found to be involved in bolting and flowering regulation (Wang et al., 2007; Kumimoto et al., 2008; Zhu and Helliwell, 2010).

**Figure 5:**
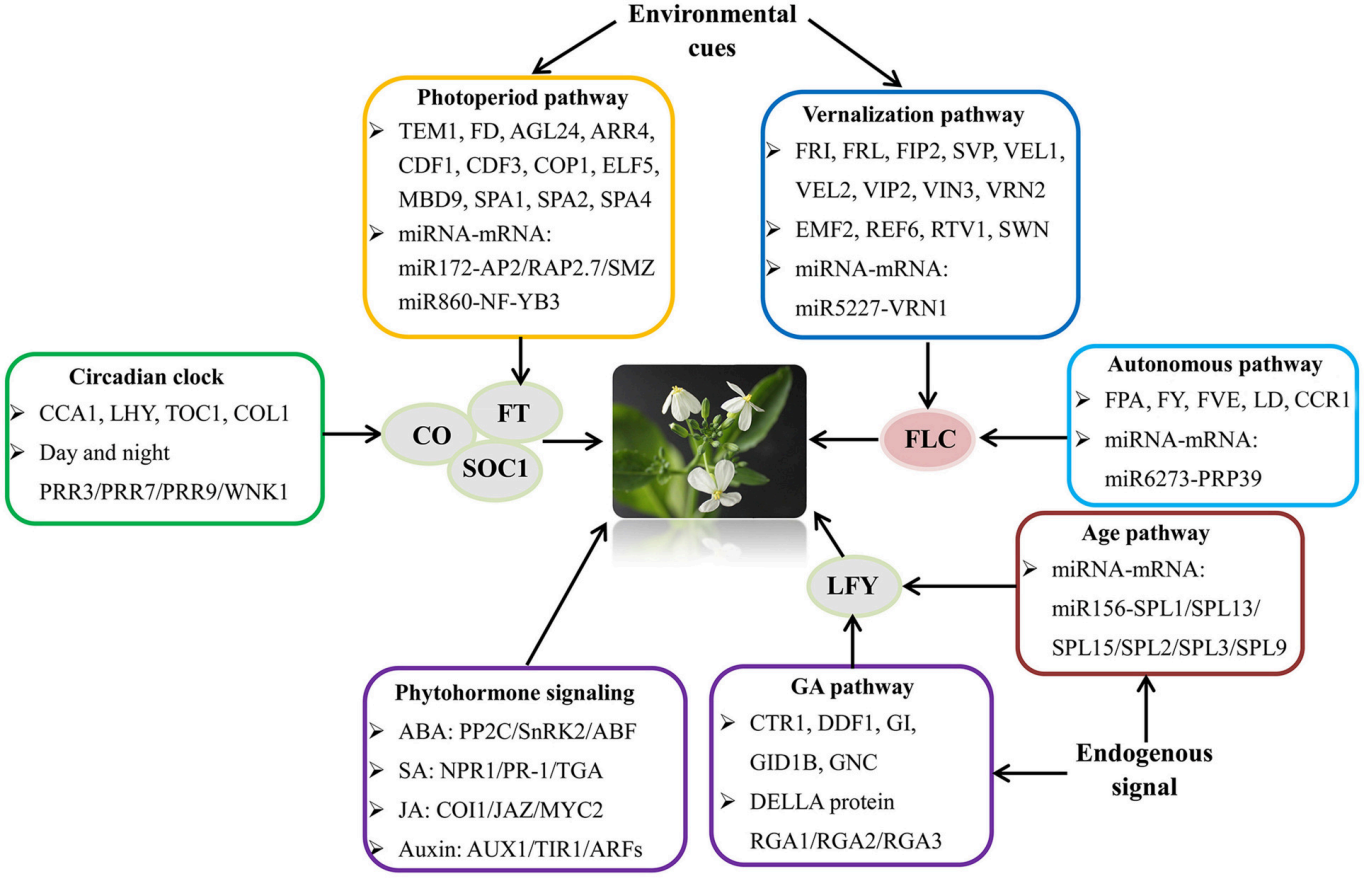
**The putative model of flowering regulatory network integrating various flowering pathways and transcriptional processes in radish**. The *FT, SOC1, LFY, FLC*, and *CO* genes were flowering pathway integrators.

To gain insights into the bolting and flowering regulatory network in radish, a putative model for summarizing the bolting and flowering-related DEGs and miRNAs was proposed (Figure 5). The critical genes involved in various flowering pathways and phytohormone signaling pathways were displayed in the schematic regulatory network of radish bolting and flowering. According to the known *Arabidopsis* flowering regulatory network (Fornara et al., 2010; Srikanth and Schmid, 2011), we speculated that the transcriptional regulations of several floral integrators including *FT, CO, SOC1, FLC*, and *LFY*, could integrate the signals from various pathways and modulate the radish bolting and flowering (Figure 5). Moreover, the models of miR172-*AP2* and miR5227-*VRN1* have been shown to be important participants in the regulatory network of bolting and flowering (Wang et al., 2007; Zhu and Helliwell, 2010; Nie et al., 2015).

## Discussion

Radish bolting and flowering are integral stages in its complete life cycle. The timing of bolting and flowering is coordinately regulated by various endogenous and environmental signals integrating into a complexity of flowering regulation (Amasino and Michaels, 2010; Srikanth and Schmid, 2011). Recent advances in flowering genes and regulatory networks have greatly enhanced our knowledge of molecular basis underlying bolting and flowering-time control in Brassicaceae crops. However, no studies on comprehensive identification of DEGs related to radish bolting and flowering have been reported, and the regulatory mechanism of bolting and flowering-time control remains largely unexplored in radish. In this study, two cDNA libraries from leaves of radish advanced inbred line ‘NAU-LU127’ at vegetative and reproductive stages were constructed, respectively. A list of DEGs related to phytohormone signaling and transition from vegetative growth to bolting and flowering were identified and comprehensively profiled.

### The roles of plant hormone signaling in bolting and flowering

Plant hormones are endogenously occurring compounds that regulate multiple aspects of plant growth and development including flowering time (Davis, 2009; Santner and Estelle, 2009). Various phytohormones have been implicated in the developmental transition of flowering (Davis, 2009; Domagalska et al., 2010). The pathways of several hormones including auxin, GA, ABA, SA, and JA signaling were significantly enriched by pathway-based analysis in our study (Table 3).

GA pathway is one of the genetic flowering pathways, which could interact with several pathways and is integrated into the flowering regulatory complexity (Srikanth and Schmid, 2011). The role of GA pathway in flowering time has been thoroughly investigated in *Arabidopsis* and several fruit trees (Wilkie et al., 2008; Mutasa-Göttgens and Hedden, 2009). Many genes related to GA metabolism and signaling were involved in GA-mediated regulatory process of flowering (Mutasa-Göttgens and Hedden, 2009; Domagalska et al., 2010). GA exerting its biological functions on floral transition and development is mainly dependent on the growth inhibitor DELLA proteins (Mutasa-Göttgens and Hedden, 2009). GA signaling promotes flowering through initiating the degradation of transcriptional regulator DELLA and activating the expression of *SOC1, AGL24* and *LFY* (Davis, 2009; Mutasa-Göttgens and Hedden, 2009). As expected, both the decreased transcript abundance and expression level of *DELLA* (CL7599.Contig1) were detected in radish reproductive stage compared with vegetative phase (Figure 4). The ABA pathway, which is antagonistic to GA, has been demonstrated to delay flowering through modulating DELLA activity and affecting the transcriptional expression of floral repressor *FLC* (Achard et al., 2006; Domagalska et al., 2010). In the current study, unique transcripts annotated as *PP2C* and *ABF*, ABA signaling components, were identified and differentially regulated during radish bolting and flowering (Figure 3), which is consistent with the results in litchi (Zhang et al., 2014c) and soybean (Wong et al., 2013). These findings suggested that the differential expressions of ABA signaling-related genes may be associated with the timing of radish transition to bolting and flowering.

Function of SA in accelerating transition to flowering is pronounced by SA-deficient mutants of *Arabidopsis* (Martínez et al., 2004). SA could negatively regulate the floral repressor *FLC* and activate the flowering promoter *FT* which strongly highlights the positive role of SA in flowering transition (Martínez et al., 2004). SA promotes the activation of NON-EXPRESSOR OF PR-1 (NPR1) proteins, whose interaction with TGA transcription factors could induce the expression of PR genes (Wu et al., 2012). Moreover, JA is also implicated in flowering regulatory process and delays flowering in *Arabidopsis* (Krajnčič et al., 2006; Riboni et al., 2014). JA signaling pathway has been involved in three molecular elements including JA receptor gene *COI1*, transcriptional repressor JAZ protein and some transcription factors, e.g., the bHLH family (Krajnčič et al., 2006). Notably, recent studies have demonstrated the regulatory role of *COI1* in delaying flowering mediating the repressed expression of *FT* (Zhai et al., 2015). In this study, some transcripts belonging to the main components of SA and JA signaling were found, including *NPR1, TGA, PR, JAZ, COI1*, and *MYC2* (Table 3). In addition, previous studies reveal that auxin is necessary for flower initiation and floral organ identity (Cheng and Zhao, 2007). We also found the critical genes related to auxin signaling such as *AUX1, SAUR, TIR1*, and *ARFs* (Table 3; Table S4). Overall, these results reveal that phytohormone-mediated transcriptional reprogramming are crucial to the transition of bolting and flowering and participate in its regulatory network of radish. The characterization of critical genes in plant hormone signaling pathways would greatly help to illuminate the complex genetic network of bolting and flowering in radish.

### The complex bolting and flowering regulatory network in radish

Multiple genetic flowering pathways integrating endogenous and environmental signals determine the transition from vegetative growth to reproductive development. Studies in *Arabidopsis* have revealed the participation of more than 200 flowering-related genes in the intricate regulatory network (Fornara et al., 2010; Srikanth and Schmid, 2011). In this study, 95 candidate genes related to bolting and flowering were isolated and involved in five major flowering pathways within genetic regulatory network (Table S5; Figure 5). It is inferred that known genetic pathways and critical flowering genes may conservatively present in radish, being consistent with the reports in maize (Dong et al., 2012), soybean (Jung et al., 2012), and citrus (Zhang et al., 2011). Gene expression profiling revealed that these genes were differentially expressed between NAU-VS and NAU-RS libraries, suggesting their putative important roles in radish bolting and flowering.

The complex regulatory network of *Arabidopsis* is composed of five major converging pathways (Fornara et al., 2010; Srikanth and Schmid, 2011). It is believed that endogenous developmental signals such as developmental stages of plants and phytohormones monitor flowering time through age, autonomous and GA pathways, while environmental cues regulate flowering time through the photoperiod and vernalization pathways in response to day length or temperature (Srikanth and Schmid, 2011; Capovilla et al., 2015). The signals from photoperiodic process are converted into the transcriptional regulation of key genes such as *FT, CO, AP1*, and *AP2* to affect flowering time (Kikuchi and Handa, 2009; Amasino, 2010; Srikanth and Schmid, 2011). The florigen gene *FT* as a floral integrator is central for the photoperiodic flowering pathway of long-day plant *Arabidopsis*, which is perceived in leaves and transported to the shoot apex initiating floral transition (Huang et al., 2005; Parcy, 2005). The role of *FT* in promoting flowering has been proven by mutants and overexpressed transgenic analysis in *Arabidopsis* (Amasino, 2010; Srikanth and Schmid, 2011). As expected, the homolog of *FT* (FD571044) was up-regulated in reproductive stage of radish (Figure 4), indicating that the *RsFT* gene could positively regulate the development transition of bolting and flowering (Figure 5). Under long-day condition, the *FT* expression is activated by *CO*, which is a floral activator and modulated by the circadian clock and day length (Suárez-López et al., 2001; Amasino, 2010; Johansson and Staiger, 2015). The link between circadian clock and flowering control may be mainly mediated by the transcriptional expression of *CO* (Fujiwara et al., 2008; Johansson and Staiger, 2015). In *Arabidopsis*, two essential circadian clock components *LATE ELONGATED HYPOCOTYL* (*LHY*) and *CIRCADIAN CLOCK ASSOCIATED1* (*CCA1*) function in photoperiodic flowering and regulate flowering pathway by controlling the rhythmic expression of *CO* and *FT* (Fujiwara et al., 2008). In this study, some transcripts belonging to *CO, CCA1* and *LHY* homologs were found to be up-expressed in reproductive phase with DGE sequencing and RT-qPCR analysis (Figure 4), suggesting the critical roles of these genes in the transition of radish bolting and flowering.

The vernalization and autonomous pathways converge on the flowering repressor *FLC*, and many genes involved in these two pathways could control flowering time through affecting *FLC* expression (Amasino, 2010). The high level of *FLC* delays flowering and requires its activator *FRI* (Michaels and Amasino, 1999; Choi et al., 2011). Recently, several naturally occurring spliced transcripts of *FLC* were found and isolated from *B. rapa* (Yuan et al., 2009) and orange (Zhang et al., 2009), which were proven to be associated with variations in flowering time. The transcriptional co-expression analysis in *B. rapa* indicated that *BrFLC2* may be the major regulator of flowering time in genetic flowering network (Xiao et al., 2013). In this study, we found putative homologs of *FLC* (CL1584.Contig3) from late-bolting radish, which was down-regulated in reproductive stage compared with vegetative stage, with similar patterns being detected in *FRI* and *FRL* (Figure 4). In addition, similar results were found in other homologous genes in vernalization pathway, including *FIP2, EMF2, VRN1*, and *VRN2* (Table S5). These results indicate that the genetic elements of the vernalization pathway may be of importance for the manipulation of radish bolting and flowering time.

Furthermore, miRNAs as central regulators of gene expression have been shown to be implicated in multiple genetic pathways governing flowering time (Spanudakis and Jackson, 2014; Wang, 2014). The newly defined age pathway of flowering, which is controlled by miR156 and its target *SPL* transcription factors (Wang et al., 2009b), regulates flowering time and interacts with vernalization, photoperiodic and GA pathways (Zhou et al., 2013; Spanudakis and Jackson, 2014; Wang, 2014). Several members of *SPL* family were identified in this study, including *SPL1, SPL2, SPL3, SPL9, SPL13*, and *SPL15* (Table S5). It was known that miR172 is down-regulated by the age-dependent expression of *SPL9* (Wu et al., 2009; Spanudakis and Jackson, 2014). The target genes of miR172 are a class of *AP2*-like transcription factors including *AP2, TARGET OF EAT 1-3* (*TOE1-3*), *SCHLAFMÜTZE* (*SMZ*), and *SCHNARCHZAPFEN* (*SNZ*), which act as floral repressors (Zhu and Helliwell, 2010). The levels of these *AP2*-like genes are relatively high during plant seedling stage and decline with plant development, ultimately relieving the repression of flowering to trigger flowering (Aukerman and Sakai, 2003; Zhu and Helliwell, 2010). Consistent with these evidences, the down-expressed patterns of *AP2* (CL1275.Contig1), *SMZ* (Rsa#S42015352), and *RAP2.7* (CL2600.Contig3) were detected at reproductive stage in this study (Figure 4). Moreover, correlation analysis revealed that some bolting and flowering-related DEGs were targeted by specific miRNAs forming the transcriptional model of miRNA-mRNA pairs (Table S6). These findings reveal that some miRNA-DEG models including miR5227-*VRN1*, miR6273-*PRP39*, and miR860-*NF-YB3* are crucial participators and integrated into the intricate genetic networks of bolting and flowering in radish (Figure 5).

## Conclusions

In summary, RNA-Seq technology was employed to systematically identify DEGs at transcriptome-wide level during radish transition from vegetative growth to bolting and flowering in this study. To our knowledge, this is the first investigation to illustrate the expression profiles of bolting-related genes and dissect the bolting and flowering regulatory network in radish. In this study, a total of 5922 DEGs were identified from late-bolting radish leaves. Several candidate genes related to plant hormone signal and bolting and flowering regulatory pathways were characterized and implicated in the complex networks of bolting and flowering regulation. Correlation analysis suggested that the miRNA-mRNA regulatory models played pivotal roles in determining bolting and flowering time. Moreover, a schematic regulatory network of radish bolting and flowering was put forward for characterization of DEGs and miRNAs. These results provided essential information for genetic control of radish bolting and flowering, and would facilitate unraveling the molecular regulatory mechanism underlying bolting and flowering in radish and other root vegetable crops.

## Author contributions

SN, CL, and LL designed the research. SN, XS, and MT conducted experiments. SN, LX, and YW participated in the design of the study and performed the statistical analysis. SN analyzed data and wrote the manuscript. LL and EM helped with the revision of manuscript. All authors read and approved the manuscript.

### Conflict of interest statement

The authors declare that the research was conducted in the absence of any commercial or financial relationships that could be construed as a potential conflict of interest.

## Supplementary material

Table S1 **All the identified DEGs in NAU-VS and NAU-RS libraries**.

Table S2 **GO enrichment analysis for differentially expressed transcripts with corrected *P* ≤ 0.05**.

Table S3 **KEGG pathway analysis of differentially expressed transcripts in radish**.

Table S4 **The DEGs involved in plant hormone signal transduction pathway**.

Table S5 **The DEGs involved in the transition of vegetative growth to bolting in radish**.

Table S6 **The identified DEG and miRNA pairs during radish bolting and flowering**.

Table S7 **The primers of DEGs for RT-qPCR in radish**.

Figure S1 **The identified genes involved in plant hormone signal transduction by KEGG analysis**.
