# Supplementary figures and images for: Transcriptomic Analysis Identifies Differentially Expressed Genes (DEGs) Associated with Bolting and Flowering in Radish (Raphanus sativus L.)
Source: Front Plant Sci. 2016 May 24;7:682. doi: 10.3389/fpls.2016.00682 (PMC4877535; doi:10.3389/fpls.2016.00682)

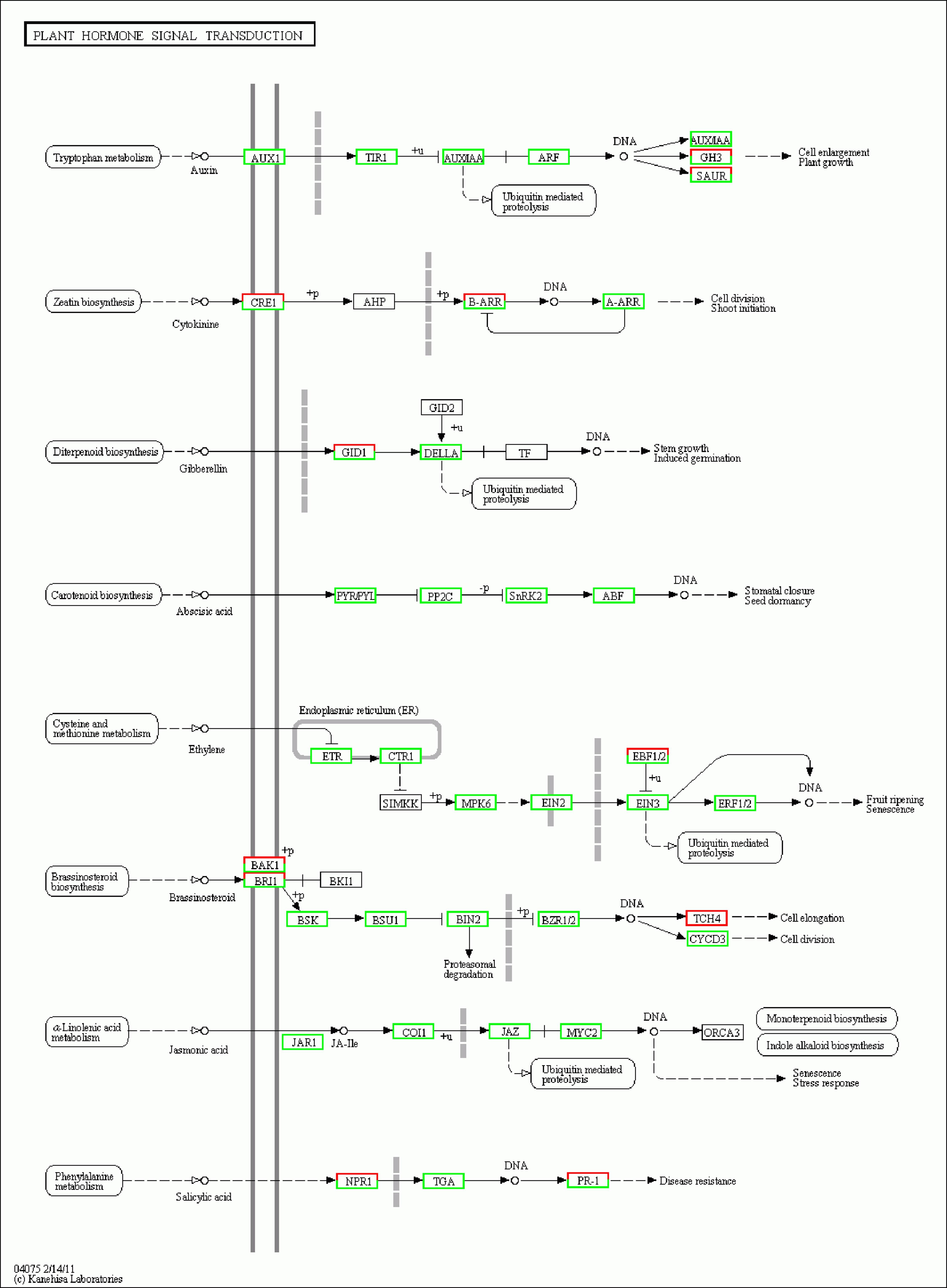

Supplement: Figure S1 — The identified genes involved in plant hormone signal transduction by KEGG analysis. [file Image1.JPEG]
